# Fluoride release from fluoride varnishes exposed to commonly consumed beverages: An *in vitro* study

**DOI:** 10.4317/jced.60022

**Published:** 2023-03-01

**Authors:** Sophia-Alejandra Casimiro-Iriarte, Lourdes-Rosa Chiok-Ocaña

**Affiliations:** 1 DDS., Master Student in Dentistry, School of Dentistry, Faculty of Sciences of Life and Health, Universidad Científica del Sur, Lima, Peru; 2 DDS. MSc. PhD., Professor Division of Dental Materials, School of Dentistry, Faculty of Sciences of Life and Health, Universidad Científica del Sur, Lima, Peru

## Abstract

**Background:**

The aim of this study was to compare the *in vitro* fluoride release from fluoride varnishes exposed to commonly consumed beverages.

**Material and Methods:**

One hundred and twenty acrylic blocks were divided randomly into ten experimental groups (n= 12 per group). For the experiment, 24 blocks were prepared for each fluoride varnish (Duraphat®, Duofluorid XII®, Clinpro™, MI Varnish™ and Profluorid®). The blocks were placed into artificial saliva for 30 minutes and in carbonated beverage or fruit juice for up to 24 hours. Artificial saliva and beverages were evaluated to determine the fluoride release using an ion-selective electrode. Data were analyzed using ANOVA F, Friedman and Kruskal Wallis test for bivariate analysis; and three-way ANOVA (fluoride varnish, beverages, exposure time).

**Results:**

When the fluoride varnishes were compared according to each exposure time, a statistically significant difference was found between all the fluoride varnishes for each evaluation time on carbonated beverage and fruit juice. MI Varnish™ presented the highest fluoride release in carbonated beverage (94.44±5.47ppm) and fruit juice (126.16±8.89ppm) at 8 hours. Duraphat® presented the lowest fluoride release at baseline (0.44±0.08ppm) for carbonated beverage group. A three-way comparison between fluoride release, exposure time and fluoride varnish were statistically significant (*p*<0.001). When evaluating the effect of the three independent variables together on fluoride release, it was found that the variables fluoride varnish (*p*<0.001) and exposure time (*p*<0.001) contributed to the release of fluoride.

**Conclusions:**

The type of fluoride varnish and the time after the application contribute to the fluoride release model.

** Key words:**Fluorides, topical, sodium fluoride, beverages.

## Introduction

Sodium fluoride is used in dentistry in various fluoride materials as a sensitivity treatment, as well as in dental caries prevention through topical applications, especially in children and teenagers. In 2018, the U.S. Food and Drug Administration (FDA) approved fluoride varnishes (FV) for anticaries treatment (1). Also, the recommendation of FV for individuals at risk of dental caries has been described in clinical practice guidelines and health care programs around the world (2-4). In addition, FV are a modality of topical fluoride, a term that describes delivery systems that provide fluoride to dental surfaces exposed to them, at high concentrations for a local protective effect (5). Since FV have an important role in caries prevention and is commonly used in dental practice, it is important to determine the concentration of fluoride that varnishes released.

On the other hand, it is highly common the massive consumed of beverages and food with acidic pH level from children and teenagers (6). Moreover, manufacturers do not mention the type of diet the patient should consumed after FV application. Manufacturer’s instructions suggest time in which food should not be consumed and the recommended hours of a soft diet however, there are no instructions related to the pH of the diet post application (7). Hence, information that describes if an acidic condition could affect fluoride release (FR) it is necessary.

The condition to which FV is exposed could affect their FR. However, while FR has been extensive studied from different varnishes, *in vitro* and *in vivo* (8-14), there is not enough information whereas if the acidic condition modifies the release (7). Moreover, there has not been studies to determine if non-alcohol beverages could affect FR, since these commercial drinks have as part of their composition citric acid, phosphoric and malic acid (15). Thus, the aim of this study was to compare the *in vitro* release of fluoride from FV exposed to commonly consumed beverages.

## Material and Methods

Details about the composition of evaluated FV and their manufacturer´s dietary instructions related to beverages are on Table 1.

**Table 1:**
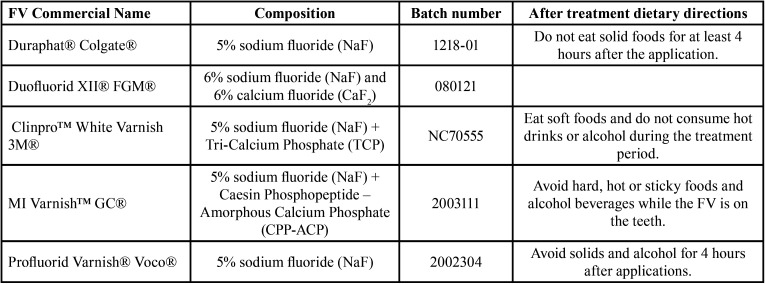
Dietary directions for the tested FV related to beverages.

For sample size calculation, the formula of compare K means 1 way-ANOVA for carbonate beverage and fruit juice was used conducted at 5 % significance level and a 95% power, from the pilot study. The pilot study was based on the final sample size of a previous study (n=3) (7) obtaining a sample of 1 specimen per group. One hundred and twenty acrylic (methyl methacrylate) blocks (25mmx25mmx9mm) were used as study substrates and divided randomly into ten experimental groups (n= 12 per group):

G1: 5% NaF (gold standard: Duraphat® Colgate-Palmolive, Hamburg, Germany) exposed to carbonated beverage 

G2: 6% NaF + 6% CaF2 (Duofluorid XII® FGM, Joinville, Brazil) exposed to carbonated beverage

G3: 5% NaF + TCP (Clinpro™ 3M ESPE™, Saint Paul, USA) exposed to carbonated beverage 

G4: 5% NaF + CPP-ACP (MI Varnish™ GC®, Tokyo, Japan) exposed to carbonated beverage

G5: 5% NaF (Profluorid® Voco, Cuxhaven, Germany) exposed to carbonated beverage

G6: 5% NaF (gold standard: Duraphat®) exposed to fruit juice

G7: 6% NaF + 6% CaF2 (Duofluorid XII®) exposed to fruit juice

G8: 5% NaF + TCP (Clinpro™) exposed to fruit juice

G9: 5% NaF + CPP-ACP (MI Varnish™) exposed to fruit juice 

G10: 5% NaF (Profluorid®) exposed to fruit juice

The blocks were cleaned with deionized water and the FV were applied in a smooth surface of each acrylic block. The amount of FV applied in each block was determined after measuring the unit-dose packages using an analytical balance (ED 224S, Sartorius GA, Göttingen, Germany). It was determined to use 40mg in each acrylic block using the manufacture’s applicator for Clinpro™ and MI Varnish™, and a similar brush for the other three FV according to the manufacturer’s instructions. For the FV in individual unit-dose package, they were thoroughly mixed prior to use with the manufacture’s applicator for ten seconds to homogenize the FV since components of sodium fluoride varnishes can separate during storage (7).

For the experiment, 24 blocks were prepared for each FV: half of the acrylic blocks for carbonated beverage (Coca-Cola®) and the other half for fruit juice (Pulp®). To measure the pH level of both beverages, a pH benchtop meter (inoLab® pH 7310, WTW™ Xylem Analytics, Weilheim, Germany) was used and the measurements were in triplicate immediately after opening at 20°C, the mean of those lectures were recorded as the pH level (15). The main characteristics of the beverages are showed in Table 2.

**Table 2:**
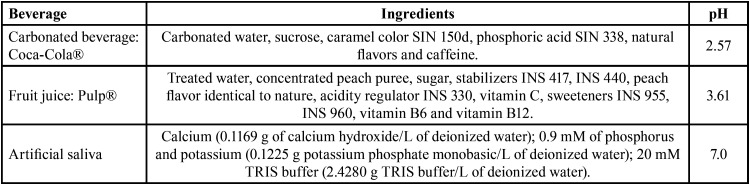
Main characteristics of the solutions.

After the FV application, each block was submerged individually in 30ml of artificial saliva in a 100ml plastic beaker (16). The beakers were placed onto an orbital shaker at 100 rpm for 30 minutes. Then, the blocks were removed from the beakers and the artificial saliva was preserved for the measure of fluoride concentration (7).

Immediately after, each block was fully submerged in the beakers with the beverages. Half of the blocks were exposed to 30ml of carbonated beverage (Coca-Cola®; pH 2.57) and the other half were exposed to 30ml of fruit juice (Pulp®; pH 3.61), the beakers were placed onto the orbital shaker at 100 rpm for 30, 420 (7 hours) and 960 minutes (16 hours). The beverages were decanted in a plastic container and renewed at 60 minutes (1 hour) and 480 minutes (8 hours) the FV application (7,13).

-Measure the fluoride release

The FR was determined using an ion-selective electrode (9609 BNWP-Orion Research Inc., Beverly, MA, USA) coupled with a benchtop meter (Orion Star A214, Orion Research Inc.). The calibration of the electrode was performed using a calibration curve with standard solutions of known fluoride concentrations: 0.1, 1,10, 40 and 100 ppm obtained from a standard solution of 100 ppm F- (Thermo Scientific Orion) (13,17).

The values for the standard curve were obtained from 750 µL of Total Ionic Strength Adjustor Buffer (TISAB II, Thermo Scientific Orion) and 750 µL of the fluoride standard solutions. The fluoride concentration was calculated using a linear regression equation between de electrical conductivity (mV) and the logarithm of the fluoride concentration of the standard solutions (10,17). A new calibration was performed before reading the solutions (mean r2=0.9999) and checked every hour with solutions of know fluoride concentrations: 1, 2 and 10 µg F-/mL (Thermo Scientific Orion) with a 2.41% of coefficient of variation between the expected and the calculated concentrations through the whole experiment.

The aliquots of each solution: artificial saliva, carbonated and fruit juice were mixed with TISAB II in a 1:1 proportion and the concentrations were obtained compared to the standard curve, by transforming the mV to ppm. The samples were read duplicate and the mean of the two reading was recorded as the fluoride concentration (ppm) (7,17).

The statistical analyses were performed using the statistical software Stata 21.0. ANOVA F test for repeated measurements and Bonferroni post-hoc test; and Friedman test and Wilcoxon post-hoc test were used for comparisons between FR and exposure time. Kruskal Wallis and U de Mann Whitney post-hoc test were used for comparisons between FR and FV. An ANOVA three-ways was used for de effect of FV, exposure time and beverages on the FR.

## Results

**Figure 1:**
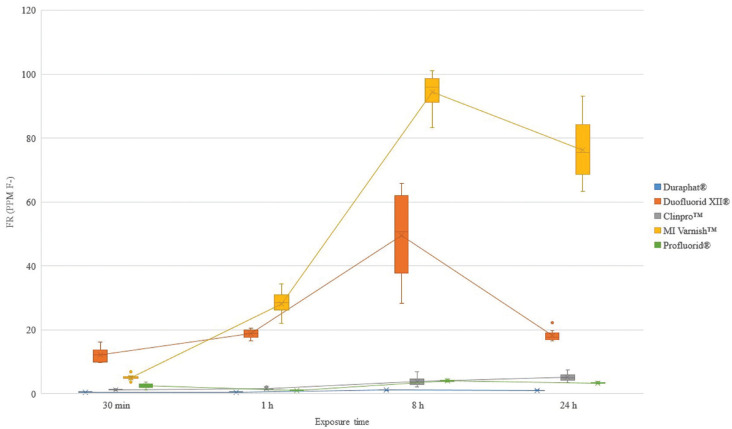
Fluoride release (ppm F-) of FV exposed to carbonated beverage.

For the carbonated beverage, the FR increased from 30 minutes at baseline on artificial saliva to 8 hours for Duraphat®, Duofluorid XII® and MI Varnish™; time after which the FR decreased. On the other hand, the FR increases numerically from 30 minutes on artificial saliva to 24 hours for Clinpro™. Profluorid® also had a different tendency, with a decrease on FR from 30 minutes on artificial saliva to 1 hour, an increased from 1 to 8 hours and a final decrease on FR to 24 hours (Fig. 1).

**Figure 2:**
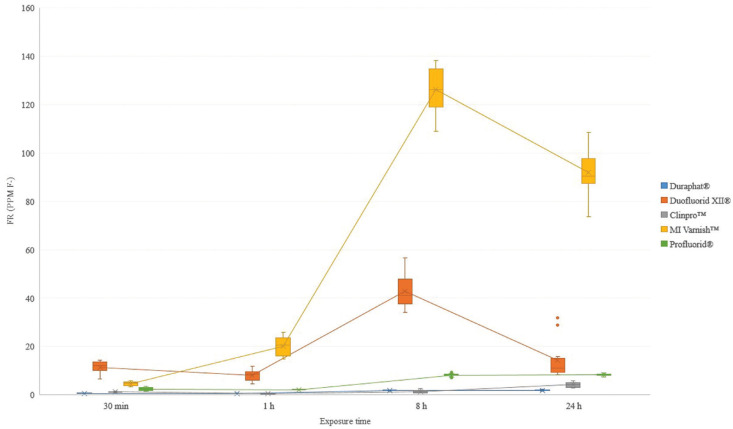
Fluoride release (ppm F-) of FV exposed to fruit juice.

On fruit juice, FR for MI Varnish™ was similar to carbonated beverage. The FV Duraphat®, Clinpro™ and Profluorid® showed a decreased on FR from 30 minutes at baseline on artificial saliva to 1 hour, then an increased was showed from 1 to 24 hours. Duofluorid XII® presented a similar tendency than the one presented by Profluorid® exposed to carbonated beverages (Fig. 2).

When the FV were compared according to each time of exposure, a statistically significant difference was found between all the FV for each of the evaluation times on carbonated beverages (Table 3) and fruit juice (Table 4). A three-way comparison between FV, exposure time and commonly consumed beverage FV was statistically significant (*p*<0.001). When evaluating the effect of the three independent variables (FV, beverages and exposure times) together on the release of fluoride, it was found that the variables FV (*p*<0.001) and exposure time (*p*<0.001) were those that contributed to the release of fluoride, whereas the type of beverage was not statically significant (*p*=0.458).

**Table 3:**
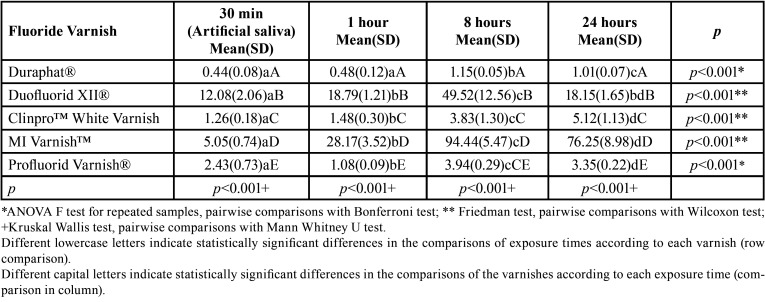
Mean (DS) of Fluoride Release (ppm F-) of tested fluoride varnishes exposed to artificial saliva at baseline and to carbonated beverage (pH = 2.57) at 1, 8 and 24 hours after the first exposure.

**Table 4:**
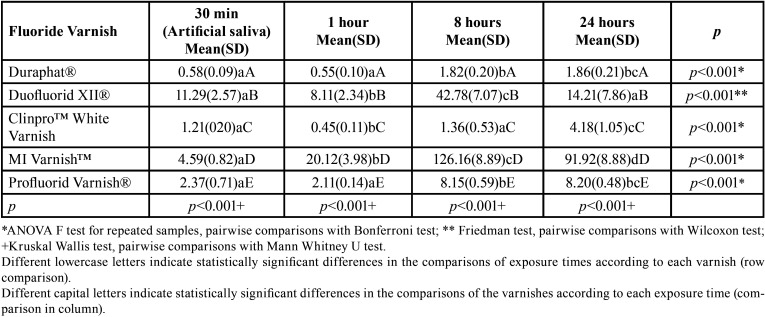
Mean (DS) of Fluoride Release (ppm F-) of tested fluoride varnishes exposed to artificial saliva at baseline and to fruit juice (pH = 3.61) at 1, 8 and 24 hours after the first exposure.

## Discussion

The present *in vitro* study shows that each FV vary its FR in artificial saliva and commonly consumed beverages depending on the exposure time. The design of the experiment was made to mimic the clinical conditions after a FV application. This was also important to define post-application indications and the clinics decision to use a specific FV according to the FR needed.

The beverages used, with a pH level of 2.57 and 3.61 for Coca-Cola® and Pulp®, respectively, did not influenced in the FR of the tested FV. On the other hand, both exposure time and type of FV, contributed to the FR model. This is similar to other studies that compared FV under a neutral pH level (8-14,17-20).

Duraphat® and Profluorid® contain 5% NaF without another active ingredient but the FR for them in both beverages was different. Furthermore, the tendency for both FV differs under carbonated beverage but a similar tendency was showed under fruit juice, even though both FV have the same type of carriers. Duraphat® contains 5% NaF with a rosin and ethanol carrier and Profluorid® is also a colophony based FV (21). So, this difference could be associated to the manufacturer’s formulation (14) of the FV and to the ingredients of the beverages.

Duraphat® presented its highest FR on carbonated beverage at 8 hours and on fruit juice at 24 hours. Despite this, Duraphat® is the FV that presented less FR overall compared to the other FV which is consistent with other studies (8-13). Duraphat® is considered the “clinical reference” because it is a commonly used FV and it has been tested in similar experiments (8). Additionally, Carvalho *et al*. described that Duraphat® exhibited the greatest preventive effect on demineralization in contrast to its low FR compared to other FV. As a result, they hypothesized that a high FR does not indicate a high cariostatic effect of the FV (13).

MI Varnish™ showed the highest FR under carbonated beverage and fruit juice, which is similar to other studies (8,20). Dehailan *et al*. found that, for total FR, MI Varnish™ released significantly more fluoride over a 24-hour period than all other FV (20). Also, Cochrane *et al*. described that the FR from MI Varnish™ was significantly higher (*p*<0.05) at 4, 24, 72 and 168 hours’ time points when compared with other FVs tested (8). This FV has as part of its formula casein phosphopeptide – amorphous calcium phosphate (CPP-ACP) that contains protein and phosphate, which are essential for reducing the solubility of calcium fluoride in aqueous solutions (22), such as the ones in this experiment.

Moreover, the highest FR for MI Varnish™ was at 8 hours in both carbonated beverages and fruit juice. Cochrane *et al*. considered this a fast release of fluoride ions. The authors attributed the fast FR to the high water solubility of CPP-ACP complexes (8).

Duofluorid XII® presented the second highest FR in carbonated beverage and fruit juice. This could be associated with its calcium fluoride and sodium fluoride composition. Cochrane *et al*. described that all tested calcium-containing varnishes in their study have a cumulative release of fluoride ions at 24 hours that was similar or better than Duraphat® (8). Another reason could be the viscosity of the FV. For instance, Carvalho *et al*. found that Duraphat® was more viscous than Duofluorid XII®, so Duofluorid XII® could be spread over a larger area of acrylic blocks. This increased their surface area in contact with the surrounding water and possibly led to greater FR (13).

The fluoride strength of all the tested varnishes was 22 600 ppm F-. All the same, the maximum fluoride release was for MI Varnish™ at 8 hours in carbonated beverage (94.44±5.47 ppm F-) and on fruit juice (126.168.89 ppm F-) with a statistically significant decrease at 24 hours. On the other hand, Clinpro™, had their peak fluoride release at 24 hours on carbonated beverage (5.12±1.13 ppm F-). The same happened on fruit juice to Duraphat® (1.86±0.21 ppm F-), Clinpro™ (4.18±1.05 ppm F-) and Profluorid® (8.20±0.48 ppm F-). In an *in vitro* study by Chhatwani *et al*., one of the studied varnishes, Fluor Protector S, showed a peak fluoride release at time 1 (after 20 days of thermal cycling). It can be assumed that Fluor Protector can deliver a high initial level of fluoride release to the surrounding oral cavity environment, but thereafter the fluoride levels fall to a minimum (23). This is similar to our findings where the FR was statistically different for exposure time according to each varnish.

In spite of the results from FR, it cannot be concluded that this is a measure to determine cariostatic effect of a FV. This because the primary action of fluoride in caries prevention is more closely related to its presence in the fluid phases of the oral cavity, where fluoride must be constantly present at low concentrations (13). Furthermore, based on their results, Dehailan *et al*. described that FR cannot be used as a predictive measure for enamel fluoride uptake which is why one should be careful in relating FR to FV efficacy (20). Also, an in situ study by Attin *et al*. made clear that within the first day after application of a highly concentrated fluoride varnish (Duraphat® - KOH soluble) fluoride deposition in close vicinity to the fluoridation occurs. This means that the preventive action of fluoride varnish is limited to the area to which varnish has been applied (24).

Lippert concluded in a previous *in vitro* study that the pH level of the solutions in which FV were submerged affects the FR. According to this study, some FV were prone to low pH fluoride loss compared to FR in artificial saliva (pH level = 7.0) (7). However, in the present study the beverages used, with a pH level of 2.57 and 3.61, did not influenced in the FR of the tested FV. This could be related to the solutions used, Lippert used anhydrous citric acid with pH levels of 2.27 y 3.75 (7), while we used commonly consumed commercial beverages. The ingredients of the beverages, such as phosphoric acid and acidity regulators, could also influenced in this difference as well as the exposure time evaluated.

Clinically, FV post-application instructions advice to not brush the teeth surface for approximately 24 hours, which is the main reason why we decided to evaluate for this long period of time. Additionally, Carvalho *et al*. decided to study clinically relevant periods of time, with intervals of 1, 8 and 12 hours because they described that FV only remains in contact with enamel for a few hours. However, they also decided to include other time points to determine whether FV could release fluoride for longer periods (13). In the same way, Dehailan *et al*. carried out an *in vitro* study in 14 FV and determined the FR on artificial saliva for a prolonged period of 24 hours in order to a better simulation of the clinical situation (20).

Bezerra *et al*. found in their *in vitro* study of different fluoridated products that a higher concentration of fluoride in saliva was observed one hour after the application. The concentration of fluoride increased over time (1- 48 hours), whilst the fluoride release tax decreased (17). On the other hand, Carvalho *et al*. observed that the highest FR occurred during the first 8 hours after application, and the amount of FR from the tested varnishes containing CaGP was significantly greater than that from the commercial fluoride varnishes studied. However, they found that after the first 8-h period, the amount of FR from these varnishes substantially decreased (13). Virupaxi *et al*., conducted *in vitro* research for 6 months and found that in the 1st week Clinpro™ XT Varnish released 18.78±7.35 ppm F- which gradually decreased to 9.78 ± 4.11ppm F- after 6 months. The FR from this varnish was reduced but still, it maintained a constant release of fluoride till 6 months in contrast to Fluoritop SR which showed the highest fluoride release for 1st week 66.92±16.30 ppm F- which rapidly decreased to 0.61±0.36 ppm F- at the end of 6 months (12).

From those results, it is concluded that the recommended time between fluoride applications will depend on the fluoride varnish. Furthermore, according to our findings, the type of fluoride varnish contributes to the fluoride release model of the fluoride varnishes (*p*<0.001).

Downey *et al*. conducted an *in vivo* study of FR from varnishes with different compositions (5% NaF, 5% NaF + TCP and 5% NaF + ACP) in unstimulated saliva. The authors attribute the variation of the FR of the varnishes to the differences in formulation (18). Also, Dehailan *et al*. carried out an *in vivo* study to investigate the concentrations of fluoride in biofilm, centrifuged and whole saliva after a single application of three 5% NaF varnishes (19). From the results of these studies, it is concluded that the formulation of other ingredients in FV can affect the concentration of fluoride in saliva which agrees with our findings in artificial saliva.

However, despite our effort to mimic the clinical scenario we cannot over interpret the results; because, as Bezerra *et al*. described, the dynamics of human saliva may interfere with the release of fluoride, as well as other physiological conditions inherent to humans, such as swallowing, chewing and body temperature (17). Likewise, Manarelli *et al*. defined as the main limitation of their *in vitro* study the evaluation of the dynamics of human saliva or pH fluctuations in the oral environment affecting the rate of release (14).

Low pH beverages, such as carbonated beverages and fruit juices, are highly consumed, especially by children and teenagers (15,25,26). That is why it is necessary a better understanding of FR from FV under various acidic conditions. Despite, the lack of statistically difference in this study, more experimentations must be done to determine if other formulas or a bigger difference between the pH level of the beverages might modify the outcomes. Furthermore, fluoride can be toxic only in highly elevated concentrations (5mg/kg) (27) for this reason, FV does not pose a risk of toxicity (7). However, the fluoride present in other products such as toothpaste, juices, cereal, salt and more (28) should also be taken into consideration in the risk of fluoride exposure. Though, it is necessary to determine if beverages different from the ones tested could affect the FR. This is essential, since, undoubtedly, it will be the patient who will ingest these concentrations. In addition, excessive fluoride intake has been linked to detrimental health effects such as the development of dental and skeletal fluorosis, increased bone fractures, and deficits in cognitive development in children (28-30).

## Conclusions

The present study shows that the *in vitro* FR had a significant difference related to each type of FV and exposure times. Thus, it is concluded that the type of FV and the time after the first FV application contribute to the fluoride release model. Nevertheless, this study presents limitations due to the fact that it is an *in vitro* experimentation and it only intended to mimic what occurs clinically. For this reason, clinical studies are necessary to confirm the results of this *in vitro* research.
